# Nutritional Properties and Antinutritional Factors of Corn Paste (*Kutukutu*) Fermented by Different Strains of Lactic Acid Bacteria

**DOI:** 10.1155/2015/502910

**Published:** 2015-05-19

**Authors:** Tchikoua Roger, Tatsadjieu Ngouné Léopold, Mbofung Carl Moses Funtong

**Affiliations:** ^1^National School of Agro-Industrial Sciences, Ngaoundere, Cameroon; ^2^University Institute of Technology, University of Ngaoundere, Ngaoundere, Cameroon

## Abstract

The aim of this study is to reduce antinutritional factors and to improve the nutritional properties of *Kutukutu* during fermentation with Lactic Acid Bacteria (LAB). For that, *Kutukutu* (700 g) was prepared in the laboratory and inoculated with pure cultures of LAB (10^9^ CFU/mL). Then, preparation was incubated for 120 h. Every 24 h, *Kutukutu* were collected, dried at 45°C for 24 h, and analyzed. The results showed that* Lactobacillus brevis* G25 increased reducing sugars content to 80.7% in *Kutukutu* after 96 h of fermentation.* Lactobacillus fermentum* N33 reduced the starch content to 73.2%, while* Lactobacillus brevis* G11,* L. brevis* G25, and* Lactobacillus cellobiosus* M41 rather increased the protein content to 18.9%. The bioavailability of Mg and Fe increased, respectively, to 50.5% and 70.6% in the *Kutukutu* fermented with* L. brevis* G25.* L. plantarum* A6 reduced the tannin content to 98.8% and* L. buchneri* M11 reduced the phytate content to 95.5%. The principal component analysis (PCA) shows that, for a best reduction of antinutrients factors and improvement of protein content and minerals, *Kutukutu* must be fermented by* L. brevis* G25 and* L. fermentum* N33, respectively. These starter cultures could be used to ameliorate nutritional proprieties of *Kutukutu* during the fermentation.

## 1. Introduction

The production of fermented corn paste by natural fermentation of grains soaked in water and ground is an artisanal transformation process of maize commonly used in Africa [1]. Such fermented corn paste can take many denominations in different countries. In Nigeria, for example, the fermented paste is called “Ogi” while in South Africa the term commonly used is “Mawe” [2]. In Cameroon, particularly in North Region, they call it “*Kutukutu*” [3]. This* Kutukutu *has an important place in the sociocultural and nutritional plan. In the sociocultural plan,* Kutukutu *is taken regularly during fast periods and is frequently used as complementary foods for infants [4]. In Cameroon, 70% of mothers give porridge prepared with* Kutukutu* to infants during the weaning period [5]. Moreover, it is a major source of proteins, carbohydrates, and calories in the diets of large number of population [6]. However* Kutukutu* contains many antinutritional factors such as phytic acid, polyphenols, and tannins which reduce bioavailability and digestibility of proteins and carbohydrates through formation of complex with minerals and inhibition of enzymes [7]. The technological processes such as mechanical, thermal, chemical, and biological processes are used to reduce antinutritional factors content and to improve the bioavailability of nutriments. Unlike thermal, chemical, and mechanical processes which can deteriorate quality of food, fermentation is one of the processes that decreases the level of antinutrients in food grains and increases the starch digestibility, protein digestibility, and nutritive value [4]. Among the microorganisms used in food fermentation, the LAB represents the principal group found on various substrates [8]. LAB are a large group of closely related bacteria that have similar properties such as lactic acid production, which is an end product of the fermentation. This LAB group includes* Lactobacillus, Lactococcus, Streptococcus,* and* Leuconostoc *species. Lactic fermentation is a common way of preparing traditional fermented food in Africa like maize porridge, alcoholic beverages, and dairy products. Several studies reported that LAB improve the nutritional quality of  foods during fermentation by increasing the protein content, reducing sugar content, reducing the antinutritional factors (phytates, tannins, and polyphenols), improving the bioavailability of minerals [9], and increasing the energy density by hydrolyzing starch into simpler compounds such as glucose and fructose [10]. Although natural fermentation improves nutritional value and organoleptic qualities of foods [9], it has a major problem of fluctuation in the quality of different foods obtained [11]. Indeed, the spontaneous fermentation process that is carried out by the development of epiphytic microflora can lead to undesirable products on the organoleptic, microbiological, or toxicological quality [11]. That is why the natural fermentation is often the main cause of diarrhea and malnutrition in children [12].

To solve this problem, there is a crucial need to isolate and identify LAB with specific physiological and metabolic properties, which can be used as starters in view to improve general food quality and nutritional value as suggested by few authors [13–17]. The aim of this study is to reduce antinutritional factors and to improve the nutritional properties of* Kutukutu* during fermentation with* L. brevis* G11,* L. brevis *G25,* L. buchneri* M11,* L. cellobiosus* M41,* L. fermentum* N33,* Lactobacillus fermentum* N25, and* L. plantarum* A6.

## 2. Materials and Methods

### 2.1. Starters

The* Kutukutu *was obtained after individual fermentation with seven LAB under laboratory conditions. The strains like* L. brevis* G11,* L. brevis *G25,* L. buchneri* M11,* L. cellobiosus* M41,* L. fermentum* N33, and* L. fermentum* N25 were isolated from fermented corn and* Kutukutu *sampled in Northern Cameroon (Maroua, Garoua, and Ngaoundere).* L. plantarum* A6 was kindly provided by the Microbiology Laboratory of CIRAD Montpelier, France.

These lactic starters stored at 4°C on agar slants were cultured by streaks on MRS agar and incubated anaerobically at 30°C for 72 h. The perfectly insulated colonies were inoculated in test tubes containing 10 mL of MRS broth and incubated at 30°C for 16 h. The resulting preparation was centrifuged at 3000 rpm for 10 min and the resulting pellet was washed in 10 mL of physiological peptone water (peptone 1 g in saline solution (0.85% NaCl), pH 7.2) and centrifuged again. The pellet obtained was suspended in 10 mL saline water. The concentration of viable cells was adjusted at 10^9^ CFU/mL using McFarland Standard tube number 4.

### 2.2. Production of* Kutukutu*

In order to evaluate the influence of LAB on the nutritional properties of the* Kutukutu* during fermentation with starters, the* Kutukutu *was produced under laboratory conditions following the traditional process with some modifications. Dry corn purchased from a local market in Ngaoundere (Adamaoua, Cameroon) was decontaminated in sterile distilled water containing benzoic acid 6% (w/v) (E210) for 24 h at room temperature. Then sterile corn was soaked in sterile distilled water for 48 h at room temperature. Grinding was proceeded after the determination of the water content (39.6%) using a metallic grinding mill. The paste obtained was mixed (1/3 w/v) with sterile distilled water and sieved through a sieve of mesh 200 *μ*m. After decantation for 24 h at room temperature, the paste was collected (water content 73%) in a sterile container and kept for inoculation and fermentation.

### 2.3. Fermentation of* Kutukutu*

**Figure 1:**
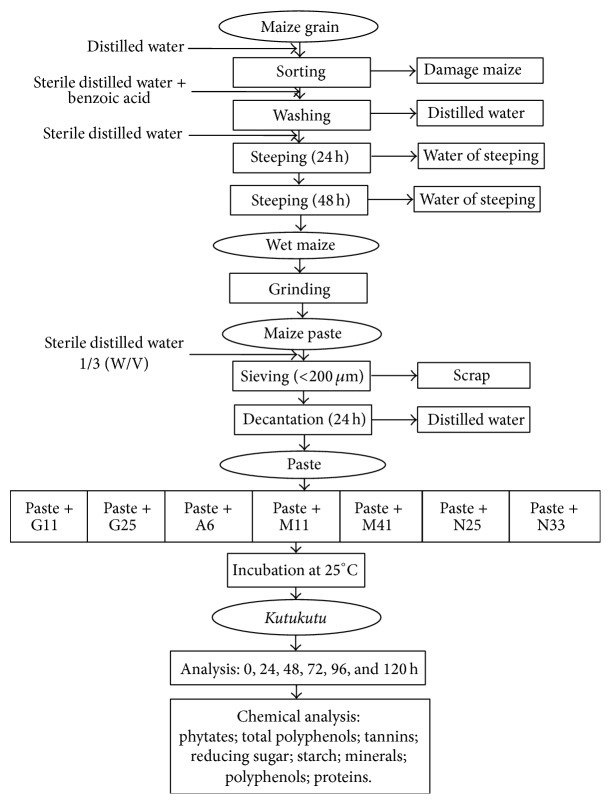
Diagram of inoculation of* Kutukutu* with LAB (G11 =* L. brevis* G11; G25 =* L. brevis* G25; A6 =* L. plantarum* A6; M11 =* L. buchneri* M1; M41 =* L. cellobiosus* M41;* L. fermentum* N33;* L. fermentum* N25).

Flasks containing 700 g of previously described paste were inoculated separately with 1 mL containing 10^9^ CFU of* L. brevis* G11,* L. brevis* G25,* L. buchneri* M11,* L. cellobiosus* M41,* L. fermentum* N11,* L. fermentum *N25, and* L. plantarum *A6. These flasks were covered and kept at 25°C for 120 h. The preparations were then homogenized on daily basis to enhance the distribution of bacteria in the medium. Aliquots were collected every 24 h, dried at 45°C for 24 h, and analyzed. The control sample was the same paste without LAB. Diagram of inoculation of* Kutukutu *with LAB in laboratory is reported in Figure 1.

### 2.4. Changes of Physicochemical Parameters in* Kutukutu*

To assess the physicochemical parameters, the pH was measured according to the method described by Afoakwa et al. [18]. The lactic acid content was determined by titration according to Obadina et al. [19] and was expressed in grams of lactic acid per 100 g of sample.

Reducing sugar was determined by the method described by Fischer and Stein [20] and the optical densities were read at 540 nm. The standard curve was drawn using a prepared aqueous solution of maltose.

The starch was determined by Jarvis and Walker method [21]. The optical densities were read at 580 nm. Standard curve was obtained using an aqueous solution of starch.

The total nitrogen content (N × 6.25) was determined after digestion of the samples according to the Kjeldahl method described by AFNOR [22] and the coloration was determined by the method of Devani et al. [23]. Standard curve was obtained using a solution of ammonium sulfate.

Minerals like iron (Fe), potassium (K), manganese (Mn), magnesium (Mg), zinc (Zn), copper (Cu), calcium (Ca), and sodium (Na) were determined by atomic absorption spectroscopy (Benton et al.) [24]. The phosphorus was determined using ammonium molybdate complex method described by Murphy and Riley [25].

The phytates content was determined by the colorimetric method described by Vaintraub and Lapteva [26], modified by Gao et al. [27], and the optical densities were read at 500 nm using a spectrophotometer. Standard curve was obtained using a solution of phytic acid.

The total polyphenols content and tannins were determined by the method of Marigo [28]. The optical densities were read at 725 nm. The formula below was used to determine the tannin content:

(1) Tannin  mg/100 DM=Total Polyphenols  mg/100 DM−Nontannin polyphenols  mg/100 DM.

### 2.5. Statistical Analysis

The results were analyzed using Statgraphics 5.0 (1998) software for the analysis of variance (ANOVA), calculation of averages, and standard deviations. Differences between means were tested using the Duncan Multiple Range Test. Sigma plot 11.0 software was used to draw the curves.

## 3. Results and Discussion

### 3.1. Changes in pH

**Figure 2:**
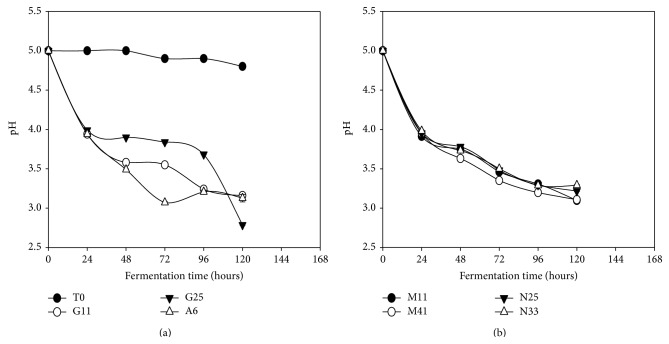
Evolution of pH in the* Kutukutu* during fermentation by the various LAB at 25°C (T0 = control; G11 =* L. brevis* G11; G25 =* L. brevis* G25; A6 =* L. plantarum* A6; M11 =* L. buchneri* M1; M41 =* L. cellobiosus* M41; N33 =* L. fermentum* N33; N25 =* L. fermentum* N25).

Generally, the pH of* Kutukutu *fermented with the different LAB trains decreased with time compared to the control (Figure 2). However,* Kutukutu *fermented with* L. brevis* G25 had the lowest pH (2.7) after 120 h. The decrease of pH is due to hydrolysis of carbohydrates during the fermentation which was followed by the production of organic acids [11]. Studies made by Ali and Mustafa [29] showed a similar reduction of pH from 4.3 to 3.4 in the sorghum dough fermented with the lactobacilli strains (*L. fermentum, L brevis,* and* Lactobacillus amylovorus*) after 6 h at 37°C.

### 3.2. Changes in Lactic Acid

**Figure 3:**
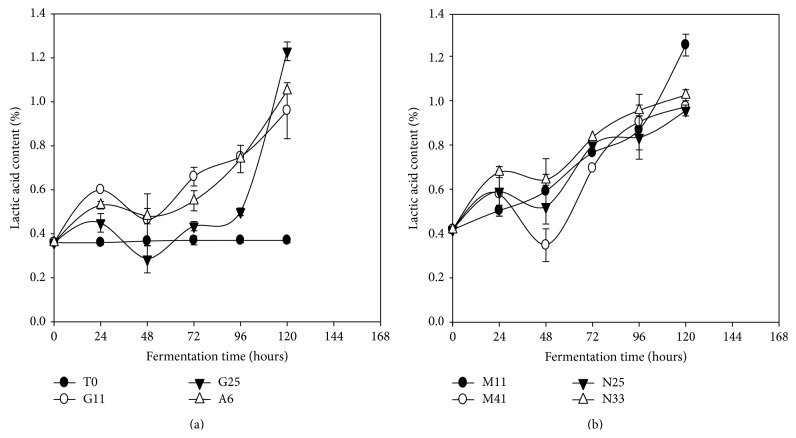
Evolution of the Lactic acid content in the* Kutukutu* during fermentation by the various LAB at 25°C (T0 = control; G11 =* L. brevis* G11; G25 =* L. brevis* G25; A6 =* L. plantarum* A6; M11 =* L. buchneri* M1; M41 =* L. cellobiosus* M41; N33 =* L. fermentum* N33; N25 =* L. fermentum* N25).

Contrarily to pH, acidity of* Kutukutu* increased significantly with time (*P* < 0.05) compared to the control (Figure 3). It was noted that* L. brevis* G25 had the highest acidity range (from 0.3 to 1.2%) during fermentation of* Kutukutu*. The increase of the acidity reflects the metabolism of sugars by LAB during fermentation [30]. From the organoleptic point of view, the acidity of* Kutukutu* makes it more appetizing for anorexic children and may also reduce bacterial contamination [31, 32]. This result is in agreement with the study of Wedad et al. [33] who showed increase in acidity of sorghum cultivar “Mugud” and cultivar “Karamaka” from 0.36 to 1.6% and from 0.36 to 1.8%, respectively, after 16 h of spontaneous fermentation at 28°C. The work of Hounhouigan et al. [34] also showed similar increase in acidity (88%) of corn flour after 72 h of fermentation.

### 3.3. Reducing Sugar

The quantity of reducing sugars increased from 0 to 48 h of fermentation and then decreased after 48 h (Table 1). An increase of 130% in reducing sugars (from 168.2 to 387.6 mg/100 g DM) of* Kutukutu* fermented with* L. buchneri* M11 after 48 h was observed. Contrarily to other LAB species, the reducing sugars were produced by* L. brevis* G25 over a long period (96 h). According to Osman [35], the increase of sugars during fermentation could be explained by the hydrolysis of starch due to amylases produced by the LAB. Osman [35] showed an increase of glucose in millet flour from 6.8 to 11.35 g/100 g after 20 h of fermentation at 30°C. Osman also portrayed an increase in fructose ranging from 1.17 to 1.20 g/100 g after 20 h of fermentation at 30°C. Reducing sugars can equally be used during the fermentation by LAB for the synthesis of various organic acids [36]. This justifies the decrease of sugars in* Kutukutu *fermented with* L. brevis *G25 (304.9 to 131.5 mg/100 g DM) after 96 h and* L. brevis* G11 (282.9 to 246.3 mg/100 g DM),* L. plantarum* A6 (263.5 to 247.6 mg/100 g DM),* L. buchneri* (387.5 to 250.7 mg/100 g DM),* L. cellobiosus* (314.1 to 210.0 mg/100 g DM),* L. fermentum* N25 (321.7 to 236.1 mg/100 g DM), and* L. fermentum* N33 (353.9 to 215.7 mg/100 g DM) after 48 h. These results corroborate with those of Osman [35] who showed reduction of glucose and fructose from 11.35 to 7.3 g/100 g and 1.2 to 0.6/100 g, respectively, for fermented millet flour between 20 and 24 h.

### 3.4. Starch

**Figure 4:**
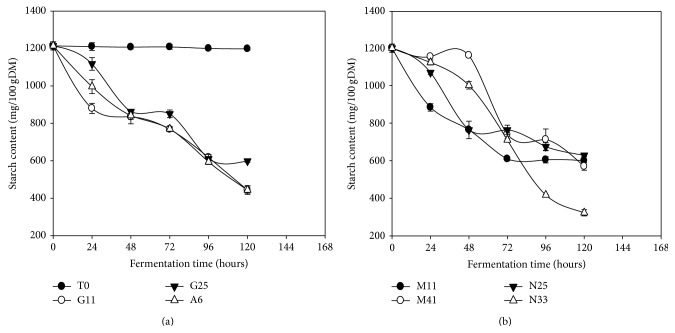
Evolution of the starch content in the* Kutukutu* during fermentation by the various LAB at 25°C (T0 = control; G11 =* L. brevis* G11; G25 =* L. brevis* G25; A6 =* L. plantarum* A6; M11 =* L. buchneri* M1; M41 =* L. cellobiosus* M41; N33 =* L. fermentum* N33; N25 =* L. fermentum* N25).

The majority of starchy compounds in the* Kutukutu* decreased significantly (*P* < 0.05) during fermentation as compared to the control (Figure 4). After 120 h of fermentation, we observed reduction of starch ranging from 1213.9 to 325.1 mg/100 g DM (73.2%) in the* Kutukutu *fermented with* L. fermentum *N33. The hydrolysis of starch by the LAB during fermentation reduces swelling of the starch granules and viscosity of the flours during the preparation of porridge [37]. The decrease of starch content in* Kutukutu* during fermentation could be due to the hydrolysis of starch due to amylases produced by the LAB into simple sugars [36]. Agati et al. [38] showed that LAB isolated from fermented maize could have a strong amylolytic activity. Hama et al. [39] showed a decrease of starch from 65.6 to 23.6 g/100 g (64.0%) after 72 h of spontaneous fermentation of* Dégué*.

### 3.5. Crude Proteins Content

**Figure 5:**
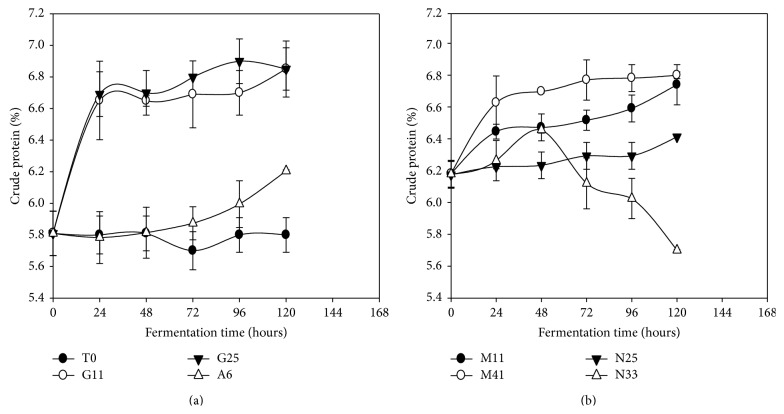
Evolution of the crude proteins content in* Kutukutu* during fermentation by the various LAB at 25°C (T0 = control; G11 =* L. brevis* G11; G25 =* L. brevis* G25; A6 =* L. plantarum* A6; M11 =* L. buchneri* M1; M41 =* L. cellobiosus* M41; N33 =* L. fermentum* N33; N25 =* L. fermentum* N25).

A slight increase of the crude proteins content was observed during the fermentation of* Kutukutu *with all selected strains excepted for* L. fermentum* N33 (Figure 5). After 120 h of fermentation, crude proteins content in* Kutukutu* fermented with* L. brevis *G11,* L. brevis *G25, and* L. cellobiosus *M41 increased from 5.8 to 6.9 g/100 g DM (18.9%) for each one. However,* L. fermentum* N33 has a different behavior from the other bacteria. Initially, an increase in proteins content ranging from 5.8 to 6.3 g/100 g DM (8.6%) was observed after 48 h of fermentation, followed by a drop from 6.3 to 5.0 g/100 g DM (20%) after 120 h of fermentation.

The increase of crude proteins content could be attributed to the use of carbohydrates by LAB [35]. These results are in agreement with those of Awade et al. [40], who showed an increase in crude proteins content by 14.63% after 14 h of fermentation of corn flour.

However the decrease in proteins content observed in* L. fermentum* N33 fermented* Kutukutu *may be explained by the fact that the LAB used these proteins for their metabolic activities during fermentation [41]. Osman [35] observed a similar reduction of protein content by 4.5% after 20 h of fermentation of millet flour at 30°C.

### 3.6. Minerals Availability

During the fermentation of* Kutukutu*, a significant increase (*P* < 0.05) in minerals was observed (Table 2), but minerals content was different between all the tested bacteria strains. The highest content of Mg, Fe, and Na was registered in* Kutukutu* fermented with* L. brevis* G25 varying between 25.9 and 39 mg/100 g DM (50.5%), 9.2 and 15.7 mg/100 g DM (70.6%), and 1.2 and 1.3 mg/100 g DM (8.3%), respectively. There was also an increase in the K and P from 82.6 to 118.8 mg/100 g DM (43.8%) and from 95.1 to 138.1 mg/100 g DM (45.2%), respectively, in* Kutukutu *fermented with* L. brevis* G11.* L. fermentum *N33 and* L. brevis* G25 increased the Zn content in* Kutukutu* with values ranging from 1.1 to 1.3 mg/100 g DM (18.2%).* L. brevis* G25,* L. brevis *G11, and* L. buchneri* increased the Cu content in* Kutukutu* from 0.1 to 0.2 mg/100 g DM (100%), while only* L. brevis *G25 and* L. brevis* G11 increased the Mn content in* Kutukutu* from 0.2 to 0.4 mg/100 g DM (100%). The increment in minerals could be explained by the reduction of antinutritional substances such as phytates and phenolic compounds which form complexes with minerals [10, 42]. Eltayeb et al. [43] observed an increase of Fe and Zn from 5.8 to 5.9 mg/100 g and from 2.9 to 3 mg/100 g, respectively, in fermented millet flour of “Garira” variety after 24 h of spontaneous fermentation at 37°C. They also noticed an increase in P, Zn, and Fe content from 183.4 to 205.3 mg/100 g, 2.9 to 3.1 mg/100 g, and 6.5 to 10.2 mg/100 g, respectively, in the fermented millet flour variety “Gadarif” after 12 h of fermentation at 37°C [43].

### 3.7. Total Polyphenols

**Figure 6:**
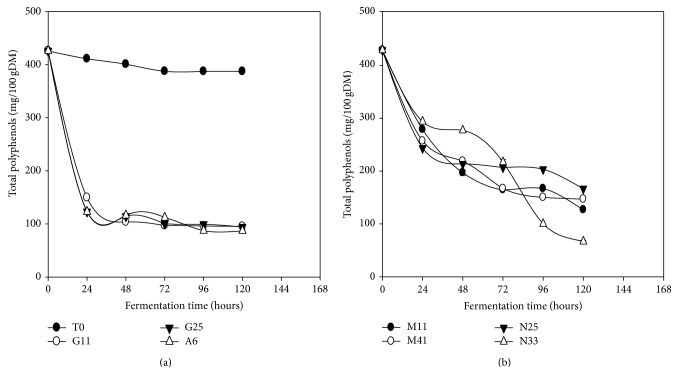
Evolution of total polyphenols in* Kutukutu* during fermentation by the various LAB at 25°C (T0 = control; G11 =* L. brevis* G11; G25 =* L. brevis* G25; A6 =* L. plantarum* A6; M11 =* L. buchneri* M1; M41 =* L. cellobiosus* M41; N33 =* L. fermentum* N33; N25 =* L. fermentum* N25).

The evolution of total polyphenols content in* Kutukutu* during fermentation is shown in Figure 6. After 120 h of fermentation, the total polyphenols content was reduced from 425.8 to 66.3 mg/100g DM (84.5%) and from 425.8 to 86.8 mg/100g DM in the* Kutukutu* fermented with* L. fermentum* N33 and* L. plantarum* A6, respectively. The reduction in polyphenols content during fermentation could be attributed to the production of polyphenol oxidases by LAB [40]. Many studies on the improvement of nutritional quality of fermented grains such as millet showed a significant reduction of the levels of polyphenols [44, 45]. Adam et al. [46] observed a reduction in polyphenols content ranging from 120.4 to 111.08 mg/100 g and from 125.1 to 107.2 mg/100 g, respectively, in millet cultivar “Ugandi” and “Dembi yellow” after 14 h of fermentation at 37°C.

### 3.8. Tannins

**Figure 7:**
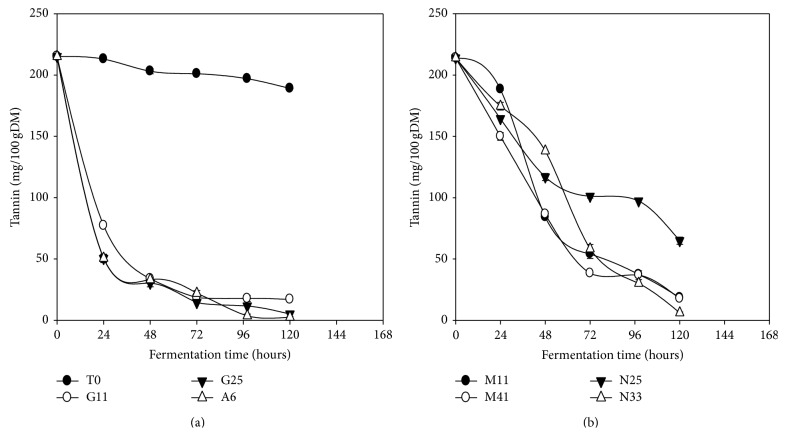
Evolution of the tannin content in* Kutukutu* during fermentation by the various LAB at 25°C (T0 = control; G11 =* L. brevis* G11; G25 =* L. brevis* G25; A6 =* L. plantarum* A6; M11 =* L. buchneri* M1; M41 =* L. cellobiosus* M41; N33 =* L. fermentum* N33; N25 =* L. fermentum* N25).

Tannins content was reduced significantly (*P* < 0.05) during the fermentation of* Kutukutu *compared to the control (Figure 7).* L. plantarum *A6 and* L. fermentum *N33 reduced the tannins content in* Kutukutu* from 215.1 to 2.5 mg/100 g DM (98.8%) and 215.1 to 4.6 mg/100 g DM (97.9%), respectively, after 120 h of fermentation. Indeed, some LAB such as* L. plantarum*,* L. pentosus,* and* L. paraplantarum* are able to degrade tannins through their acylhydrolase tannin activity [47]. This ability is often associated with the vegetable products and confers an ecological advantage to the LAB [47]. Antony and Chandra [48] showed 52% reduction of tannins in millet flour during fermentation. In the same way Onyango et al. [49] reported a significant (*P* < 0.05) reduction of tannins content after 8 days of fermentation of red sorghum flour, white sorghum and millet at 25°C.

### 3.9. Phytates

**Figure 8:**
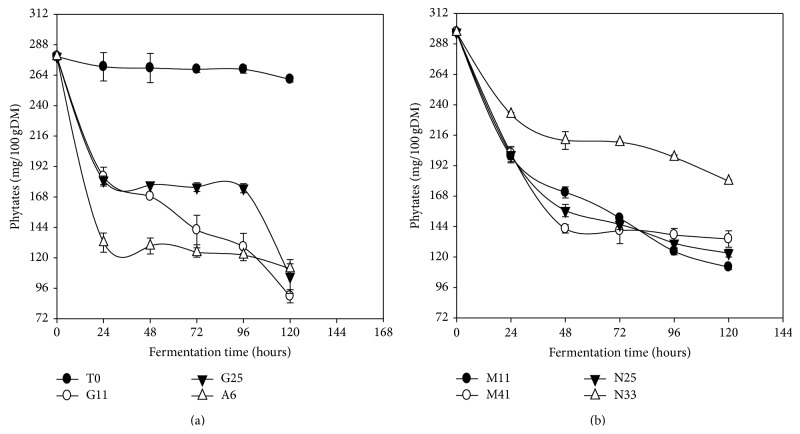
Evolution of phytates content in* Kutukutu* during fermentation by the various LAB at 25°C (T0 = control; G11 =* L. brevis* G11; G25 =* L. brevis* G25; A6 =* L. plantarum* A6; M11 =* L. buchneri* M1; M41 =* L. cellobiosus* M41; N33 =* L. fermentum* N33; N25 =* L. fermentum* N25).

The entire selected LAB reduced the phytates content after 120 h of fermentation (Figure 8). Phytates content in* Kutukutu* fermented with* L. buchneri *M11 was reduced from 278.7 to 12.4 mg/100g DM (95.5%). This observed reduction of phytates can be due to phytases and phosphatases produced by LAB which hydrolyze phytates to inositol and orthophosphates [50]. Studies made by Ejigui et al. [50] also illustrated a reduction in phytates levels ranging from 9.87 to 3.8 mg/100 g in corn flour after 96 h of fermentation at 30°C. Similarly, Onyango et al. [49] reported a significant (*P* < 0.05) reduction of phytates in red sorghum flour, white sorghum, and millet after 8 days of fermentation at room temperature. Cui et al. [51] presented a decrease of phytates to 24.3% in 4 corn cultivars during the fermentation.

## 4. Principal Component Analysis (PCA)

**Figure 9:**
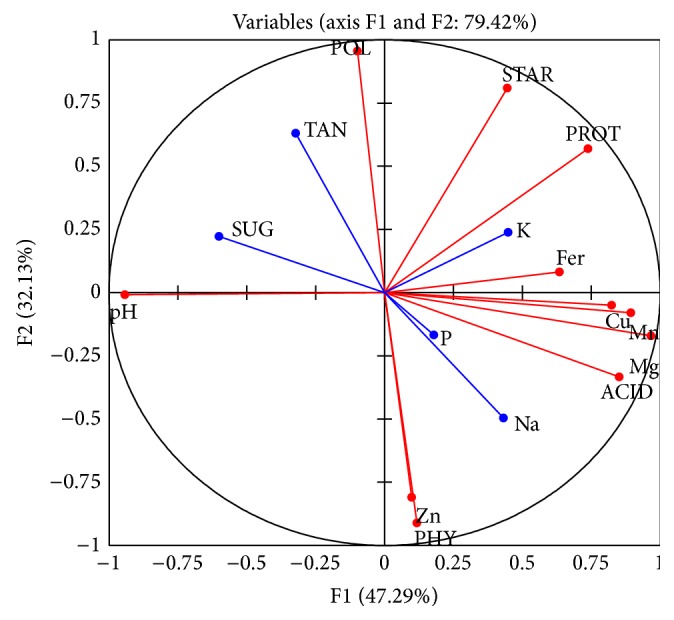
Correlation circle of the variables of* Kutukutu* in the principal component analysis axis (TAN: tannins; PHY: phytates; POL: polyphenols; PROT: proteins, ACID: lactic acid; STRA: starch; SUG: sugar) analyzed during fermentation of* Kutukutu* by LAB.

The variables used to evaluate the improvement of the nutritional quality of* Kutukutu* with LAB were attached to a principal component analysis (Figure 9). That helped to visualize correlations and to select among the 07 bacteria studied, ones who give the best results. These variables are organized in two principal components which express 79.4% of total variability. The axis F1 explains 47.29% of information and the second axis F2 explains 32.13% of information.

The analysis of the correlations between the different variables and the principal axis shows that the variables such as minerals (Mg (0.95), Mn (0.89), iron (0.63), and Cu (0.82)), proteins (0.73), lactic acid (0.85), and pH (−0.94) contribute significantly to the formation of the F1 axis, while variables such as polyphenols (0.95), phytates (−0.91), starch (0.80), and some minerals like Zn (−0.81) contribute mainly to the formation of the F2 axis. The supplementary variables (reducing sugars (−0.60)) that are classified on the axis F1 also show a significant contribution on this axis.

**Figure 10:**
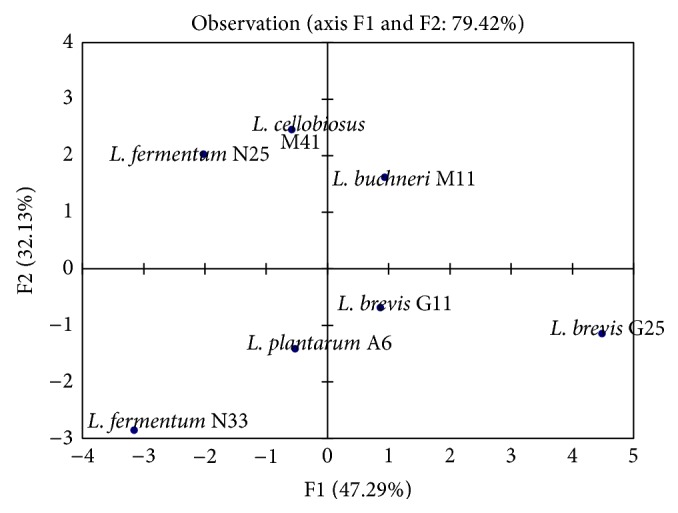
Distribution of LAB selected for the fermentation of* Kutukutu* on the axis system (F1 × F2).

When LAB are also represented in the axis system F1 × F2 (Figure 10), axis F1 corresponds to the variables induced by* L. brevis *G25,* L. fermentum* N25, while the F2 axis variables are induced by* L. plantarum* A6,* L. buchneri* M11,* L. fermentum *N33, and* L. cellobiosus* M41. However,* L. brevis* G25 (55.3%) and* L. fermentum* N33 G25 (33%) are those LAB that contribute most to the formation of this axis system (F1 × F2).

This arrangement of variables and observations on F1 and F2 axis shows that* L. fermentum* N33 helps to reduce antinutrients factors such as phytates and polyphenols, while* L. brevis* G25 contributes to increased bioavailability of minerals (Mg, Mn, Cu, and Fe), lactic acid, and protein contents.

## 5. Conclusions

The fermentation of the* Kutukutu* by selected LAB induced many changes in nutritional properties as well as antinutritional factors.* L. brevis* G25 increased (80.7%) reducing sugars content and increased the proteins content to 18.9%. It also increases availability of Mg and Fe, respectively, to 50.5% and 70.6%.* L. plantarum* A6 reduced the tannins content to 98.8% in* Kutukutu* and* L. buchneri* M11 reduced the phytates content (95.5%) in the* Kutukutu*, while, for a best reduction of phytates and polyphenols,* Kutukutu* must be fermented by* L. brevis* G25. To improve protein content and minerals (Mg, Mn, Cu, and Fe),* Kutukutu* must be fermented by* L. fermentum* N33. Both of these bacteria can be used for improving the nutritional quality of* Kutukutu* during fermentation.

## Figures and Tables

**Table 1:** Evolution of reducing sugars in the *Kutukutu* during fermentation by the various LAB at 25°C.

Evolution of reducing sugar during fermentation (mg/100 g DM)								
Time (h)	Control	G11	G25	A6	M11	M41	N25	N33
0	168.2 ± 0.0^d^	168.2 ± 0.0^b^	168.2 ± 0.0^b^	168.2 ± 0.0^a^	168.2 ± 0.0^a^	168.2 ± 0.0^a^	168.2 ± 0.0^a^	168.2 ± 0.0^a^
24	168.0 ± 0.0^d^	154.3 ± 3.5^a^	189.5 ± 2.5^c^	261.7 ± 10.5^cd^	381.7 ± 6.2^b^	277.6 ± 15.5^b^	307.0 ± 2.5^b^	286.0 ± 18.7^b^
48	160.3 ± 1.0^c^	282.9 ± 2.2^e^	291.4 ± 11.8^d^	263.6 ± 10.3^d^	387.6 ± 7.0^c^	314.2 ± 13.8^c^	321.8 ± 7.6^c^	354.0 ± 17.3^c^
72	160.2 ± 1.3^c^	270.5 ± 4.8^d^	296.1 ± 10.9^e^	254.3 ± 7.9^cd^	377.5 ± 5.2^d^	309.3 ± 12.9^d^	313.7 ± 5.2^d^	267.1 ± 17.3^d^
96	157.1 ± 2.1^b^	250.6 ± 0.6^c^	305.0 ± 9.9^ed^	234.5 ± 11.9^b^	321.3 ± 7.4^e^	287.0 ± 4.6^e^	280.9 ± 6.8^e^	218.2 ± 8.0^e^
120	149.3 ± 2.4^a^	246.4 ± 5.7^c^	131.6 ± 6.9^a^	247.7 ± 4.1^bc^	250.8 ± 0.0^f^	210.0 ± 8.8^f^	236.2 ± 9.3^f^	215.8 ± 2.5^f^

The values followed by the same letter on the same column are not significantly different (*P* > 0.05).

T0 = control; G11 = *L. brevis* G11; G25 = *L. brevis* G25; A6 = *L. plantarum* A6; M11 = *L. buchneri* M11; M41 = *L. cellobiosus* M41; *L. fermentum* N33; *L. fermentum* N25.

**Table 2:** Total minerals content in *Kutukutu* fermented with various LAB at 25°C after 120 h.

LAB	Time (hours)	Macronutrients (mg/100 g DM)					Micronutrients (mg/100 g DM)			
		Ca	Mg	K	Na	P	Zn	Cu	Mn	Fe
	**0**	**1.2 ± 0.0**	**25.9 ± 0.1** ^a^	**82.6 ± 0.0** ^a^	**1.2 ± 0.0** ^c^	**95.1 ± 0.0** ^a^	**1.1 ± 0.0** ^b^	**0.1 ± 0.0** ^a^	**0.2 ± 0.0** ^a^	**9.2 ± 0.0** ^a^

G11	**120**	1.2 ± 0.0^a^	33.6 ± 0.1^d^	118.9 ± 8.1^e^	0.5 ± 0.0^a^	138.1 ± 0.0^b^	1.2 ± 0.0^cd^	0.2 ± 0.0^b^	0.4 ± 0.0^c^	12.9 ± 0.1^e^
G25		1.2 ± 0.0^a^	39.0 ± 2.2^e^	102.1 ± 0.0^bc^	1.3 ± 0.1^c^	110.7 ± 0.0^c^	1.3 ± 0.0^e^	0.2 ± 0.0^b^	0.4 ± 0.0^c^	15.7 ± 0.0^g^
A6		1.2 ± 0.0^a^	33.8 ± 1.1^d^	112.1 ± 0.6^d^	0.8 ± 0.5^b^	132.4 ± 0.1^d^	1.2 ± 0.0^cd^	0.1 ± 0.0^a^	0.2 ± 0.0^a^	12.4 ± 0.0^d^
M11		1.2 ± 0.0^a^	34.1 ± 1.1^d^	106.6 ± 2.1^c^	0.5 ± 0.1^a^	131.0 ± 1.0^e^	1.1 ± 0.0^bc^	0.2 ± 0.0^b^	0.3 ± 0.0^b^	11.7 ± 0.1^c^
M41		1.2 ± 0.0^a^	31.7 ± 0.2^c^	105.1 ± 0.1^c^	0.4 ± 0.0^a^	118.9 ± 0.1^f^	1.0 ± 0.0^a^	0.1 ± 0.0^a^	0.2 ± 0.0^a^	14.8 ± 0.0^f^
N25		1.2 ± 0.0^a^	31.1 ± 0.4^c^	98.1 ± 0.1^b^	0.8 ± 0.0^b^	100.5 ± 0.5^g^	1.2 ± 0.0^d^	0.1 ± 0.0^a^	0.1 ± 0.0^a^	12.4 ± 0.0^d^
N33		1.2 ± 0.0^a^	28.0 ± 0.1^b^	86.0 ± 0.0^a^	0.8 ± 0.0^b^	114.3 ± 0.6^h^	1.3 ± 0.0^e^	0.1 ± 0.0^a^	0.1 ± 0.0^a^	12.1 ± 0.0^b^

The values followed by the same letter on the same column are not significantly different (*P* > 0.05).
